# Revisiting Cryptocyanine Dye, NK-4, as an Old and New Drug: Review and Future Perspectives

**DOI:** 10.3390/ijms24054411

**Published:** 2023-02-23

**Authors:** Shihui Liu, Toshihiko Matsuo, Takumi Abe

**Affiliations:** 1 Graduate School of Interdisciplinary Science and Engineering in Health Systems, Okayama University, Okayama 700-8558, Japan; 2 Department of Ophthalmology, Okayama University Hospital, Okayama 700-8558, Japan; 3 Graduate School of Medicine, Dentistry and Pharmaceutical Sciences, Okayama University, Okayama 700-8530, Japan

**Keywords:** NK-4, anti-allergic, anti-cancer, anti-inflammatory, antiviral, dilated cardiomyopathy, anti-oxidative, neuroprotective effects, cryptocyanine dye, heterocycles

## Abstract

NK-4 plays a key role in the treatment of various diseases, such as in hay fever to expect anti-allergic effects, in bacterial infections and gum abscesses to expect anti-inflammatory effects, in scratches, cuts, and mouth sores from bites inside the mouth for enhanced wound healing, in herpes simplex virus (HSV)-1 infections for antiviral effects, and in peripheral nerve disease that causes tingling pain and numbness in hands and feet, while NK-4 is used also to expect antioxidative and neuroprotective effects. We review all therapeutic directions for the cyanine dye NK-4, as well as the pharmacological mechanism of NK-4 in animal models of related diseases. Currently, NK-4, which is sold as an over-the-counter drug in drugstores, is approved for treating allergic diseases, loss of appetite, sleepiness, anemia, peripheral neuropathy, acute suppurative diseases, wounds, heat injuries, frostbite, and tinea pedis in Japan. The therapeutic effects of NK-4’s antioxidative and neuroprotective properties in animal models are now under development, and we hope to apply these pharmacological effects of NK-4 to the treatment of more diseases. All experimental data suggest that different kinds of utility of NK-4 in the treatment of diseases can be developed based on the various pharmacological properties of NK-4. It is expected that NK-4 could be developed in more therapeutic strategies to treat many types of diseases, such as neurodegenerative and retinal degenerative diseases.

## 1. Introduction

**Figure 1:**
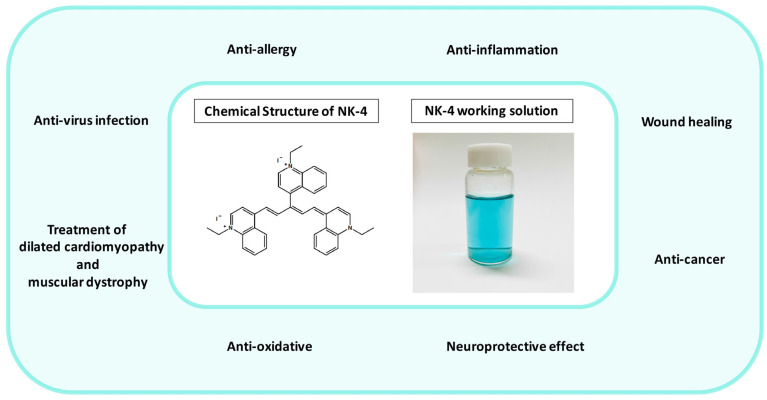
Fields of application of NK-4 (in the green circle), chemical structure of NK-4, and NK-4 working solution (0.01 mg/mL).

NK-4 (1-ethyl-4-[(1Z,3E,5E)-1-(1-ethylquinolin-1-ium-4-yl)-5-(1-ethylquinolin-4-ylidene)penta-1,3-dien-3-yl]quinolin-1-ium;iodide, IUPAC name) (Figure 1) is a divalent, cationic pentamethine trinuclear cyanine dye that consists of three quinolinium rings, short N-alkyl side chains (C2), and two iodine anions [1]. It has been studied in Japan for over 100 years and has been popularly used as an over-the-counter drug since 1951. NK-4 exhibits a variety of biological activities, such as anti-allergy, anti-cancer (inhibition of cancer cell proliferation), anti-inflammation, antiviral infection, anti-oxidative, and neuroprotective effects [2]. Additionally, it has a potential to treat dilated cardiomyopathy and muscular dystrophy [2]. In this review, we reviewed all relevant literature on NK-4 drugs.

## 2. Anti-Allergy

### 2.1. Pathogenesis and Pharmacological Therapy

Gell and Coombs’s classification divides allergies into four pathophysiological types: type I: anaphylaxis; type II: antibody-mediated cytotoxic reactions; type III: immune complex-mediated reactions; and type IV: delayed type hypersensitivity. A type I hypersensitivity is a hypersensitivity reaction that occurs within minutes after the sensitized body is exposed to the same antigen again. A type II hypersensitivity reaction is a pathological immune reaction in which IgM or IgG antibodies are combined to the corresponding antigen on the surface of target cells together with the participation of phagocytes, complement, and NK cells, leading to cell lysis or tissue damage. A type III hypersensitivity is an inflammation and tissue damage caused by the deposition of soluble immune complexes in the tissues, such as kidney, blood vessel wall, and skin, by activating the complement system; furthermore, it includes the participation of effector cells such as neutrophils and platelets, leading to cellular infiltration and localized necrosis. A type IV hypersensitivity reaction is an inflammatory reaction in which T cells are bound to corresponding antigens, leading to mononuclear cell infiltration and tissue cell damage [3]. 

A representative disease of the type I hypersensitivity reaction is allergic rhinitis (hay fever). Treatments of allergic rhinitis are, for instance, intranasal corticosteroids, oral and intranasal antihistamines, decongestants, intranasal anticholinergics, intranasal cromolyn, leukotriene receptor antagonists, combination therapy, immunotherapy, etc. [4]. 

Representative diseases of the type II hypersensitivity are Hashimoto’s disease and autoimmune hemolytic anemia (AIHA). Treatment for Hashimoto’s disease involves observation and medication, and the main therapy for Hashimoto’s disease is to control hypothyroidism, including the oral synthetic hormone levothyroxine 4 (L-T4) [5]. Prednisolone is recommended as the initial first-line treatment for primary warm AIHA. In the treatment of pathogenic B cell clones, rituximab monotherapy has become the most commonly used first-line therapy for cold AIHA. Nonpharmacological management includes thermal protection to limit hemolysis and relieve any ischemic symptoms [6]. 

The representative disease of the type III hypersensitivity is systemic lupus erythematosus (SLE). Treatments for SLE include use of immunomodulators (i.e., vitamin D and hydroxychloroquine), targeted therapy, and immunosuppressants [7].

The representative disease of the type IV hypersensitivity is allergic contact dermatitis. The primary treatment for allergic contact dermatitis is allergen avoidance. Databases such as the Exposure Allergen Management Program help patients choose allergen-free products. Treatment of acute exacerbations uses topical corticosteroids which are, however, not recommended to be used as a long-term treatment [8].

### 2.2. The Effects of NK-4

The studies investigated the immunopharmacological effects of NK-4 on type I and type IV hypersensitivity. The results demonstrated that NK-4 has a mild inhibitory effect on IgE antibody production, which is induced by heterologous passive cutaneous anaphylaxis (PCA) for 3 hours; NK-4 was shown to have a mild inhibitory effect on the homologous PCA response for 48 hours and was also demonstrated to have an inhibitory effect on the histamine release reaction by the in vitro antigen–antibody reaction in male Wistar rats (Charles River Laboratories Japan, Inc., Kanagawa, Japan). On the other hand, NK-4 significantly inhibited the cyclophosphamide (CY)-induced response from a type IV hypersensitivity reaction (delayed-type hypersensitivity (DTH)) model [1]. 

New Zealand white (NZB/W) F1 mice have been used as a model for autoimmune disease, such as SLE. NZB/W F1 mice produce autoantibodies such as natural thymocytotoxic autoantibodies (NTA) and anti-single DNA antibodies, and then develop into immune complex nephritis. The experiments show that NK-4 can significantly inhibit the level of NTA in the blood of NZB/W F1 mice while promoting the induction of suppressor T cells. On the other hand, NK-4 restored the anti-sheep red blood cells (SRBC) antibody response and anti-TNP-LPS PFC response in NZB/W Fl mice [9]. NK-4 exerts immunomodulatory effects by preventing T-cell damage and by directly activating dysfunctional B cells.

Moreover, previous studies have investigated whether NK-4 plays a regulatory role in Th2 cell activation and effector function. The results showed that NK-4 appears to selectively eliminate IL-4 and IL-5 production by Th2 cells that have been activated by antigen or anti-CD3ε monoclonal antibody. These phenomena have been accomplished by means of inhibiting the mRNA expression of the Th2-related transcription factors GATA-3 and NFATc1. On the regulation of Th2 cell effector function, NK-4 inhibits the secretion of eotaxin and TARC from IL-4/TNF-α-activated fibroblasts by inhibiting the STAT6 signaling pathway [10]. These results provide evidence for NK-4 as a therapeutic agent for Th2-mediated allergic inflammation.

## 3. Inhibiting Cancer Cell Proliferation

### 3.1. Pathogenesis and Pharmacological Therapy

Risk factors for cancer include chemicals, radiation, tobacco, excess alcohol, infections, stress, obesity, and more [11]. Cancer occurs as a series of consecutive genetic mutations that alter cellular function [12]. Cancer-related genes can promote cancer development when they are mutated, affect the cell cycle, and lead to abnormal proliferation. A tumor contains mutations in two to eight genes that promote tumorigenesis (driver genes). Driver genes can be divided into twelve signaling pathways that regulate three key cellular processes: cell fate, cell survival, and genome maintenance. Lines of evidence suggest that mutations in about 140 driver genes, such as *ABL1*, *BRCA1*, and *CDKN2A*, lead to cancer [13]. Additionally, a lack of tumor suppressor genes can lead to uncontrolled cell division [14]. From an epigenetic point of view, cancer cells are characterized by aberrant DNA methylation, which primarily targets CpG islands in regulatory elements of gene expression [15]. Past experiments have demonstrated that the expression of six genes (*CLDN3*, *DECR2*, *EVA1B*, *NTSR1*, *NME4*, and *XPNPEP2*) were highly significantly changed by alterations in DNA methylation, which can be detected by reduced representation bisulfite sequencing and RNA-seq techniques [16]. 

Near-infrared photoimmunotherapy (NIR-PIT) is a newly developed, molecularly targeted phototherapy based on the injection of a near-infrared conjugate IRDye700DX (IR700), which targets antigens expressed on the surface of cancer cells. NIR-PIT selectively destroys cancer cells, leading to immunogenic cell death, which elicits local immune responses as well as the reactivation of polyclonal CD8+ T cells against various released cancer antigens [17,18,19]. NIR-PIT not only induces immediate and highly selective cancer cell killing, but also stimulates highly effective anti-tumor immunity, thereby reducing side effects and helping patients avoid side effects associated with surgery, chemotherapy, and radiotherapy [17,18,19].

Many methods and drugs are available to treat cancer, and many more are being researched. Cancer treatment is mainly classified into local treatment, systemic treatment, and palliative care. Local treatments are used to treat cancer in specific body parts, such as surgery and radiation therapy. Systemic treatments can affect the entire body, such as chemotherapy, immunotherapy, or targeted therapy [20]. Palliative care is about improving the quality of life of patients by relieving pain and symptoms and by providing mental and psychological support [21]. 

### 3.2. The Effects of NK-4

High doses of NK-4 under light are destructive to cancer tissues, and low doses of NK-4 can effectively activate macrophages. Inflammatory lesions in photodynamic therapy with damaged and dead cancer cells which have still remained generate specific immunity, and dead cells and debris are cleared by macrophages. Studies have found that very small doses of NK-4 through photodynamic activation stimulate lymphocytes and activate macrophages, resulting in beneficial immune effects on the host organism. The activation of macrophage functions by low-dose NK-4 involves the mechanism of singlet oxygen [22]. Other studies have shown that reactive oxygen species did not participate in photodynamic cytocidal activity but that the activation of macrophages resulted from electron transfer between cationic dyes and cellular components [23]. NK-4 with an ethyl group on each quinoline structure is the most effective derivative, which can maximize the capability for the activation of macrophages. The conditions for the activation of macrophages include a 660 nm red laser or 780 nm near-infrared laser light [22,24]; a 670 nm red laser or 780 nm near-infrared laser are needed for cancer treatment by NK-4 [24,25]. 

Human lung cancers were transplanted into nude mice, and NK-4 was injected into the cancers six times after the cancers became hypertrophic. Cancers were exposed to a near-infrared laser (2 mW, 1 min) every other day for two weeks. After the first photoimmunotherapy treatment, the mice were free to drink water containing NK-4; then, the mice were allowed to drink water containing NK-4 every day. The results showed that the use of a low dose of NK-4 and laser treatment significantly enhanced the activity of macrophages, thereby increasing the immune effect. At the same time, the cancer is scarred by collagens, which have been produced from fibroblasts in the stroma [24]. Photoimmunotherapy is effective for the treatment of local deep cancer by the developed needle-type system in the presence of NK-4. At the same time, NK-4 can promote the differentiation of macrophages and lymphocytes, help healing, and improve immune function [25]. 

## 4. Anti-Inflammation and Wound Healing

### 4.1. Pathogenesis and Pharmacological Therapy

Inflammation based on time course is mainly divided into acute inflammation, subacute inflammation, and chronic inflammation. Acute inflammation occurs immediately after injury and persists for several days. Subacute inflammation is the transition from acute to chronic, lasting from 2 to 6 weeks. Chronic inflammation may persist for months or even years [26,27,28]. Acute inflammation is characterized by vasodilation, neutrophil infiltration, and fluid exudation [29]. Molecular mechanisms of inflammation are primarily initiated by the identification of characteristic molecular patterns associated with tissue damage or infection. After a generation of inflammatory response, natural innate immunity cells, such as neutrophils, macrophages, CD8+ T lymphocytes, and natural killer cells, provide an early response to noxious factors to eliminate noxious stimuli [30]. The pathological mechanisms of chronic inflammation are mainly related to stress response, adaptive immunity, and damage-associated molecular patterns [31,32,33]. 

At present, anti-inflammatory drugs mainly include non-steroidal anti-inflammatory drugs such as aspirin, indomethacin, ibuprofen, naproxen, diclofenac, celecoxib, etoricoxib, and mefenamic acid [34,35], which exert anti-inflammatory effects by inhibiting the synthesis of prostaglandins, inhibiting the aggregation of leukocytes and reducing the formation of bradykinin [36,37]. 

Wound healing includes granulation tissue proliferation, scar tissue formation, and the regeneration of various tissues. The basic process of wound healing is as follows: acute inflammation stage → cell proliferation stage → scarring stage → epidermis and tissue regeneration. There are three main types of wound healing: primary healing, secondary healing, and tertiary healing.

Inflammation is part of the physiological phase of wound healing; its purpose is to attract different immune cells to remove debris and pathogens from the wound and to create an ideal environment for the differentiation of keratinocytes and fibroblasts, which finally leads to their migration to close the wound.

The main topical medicines used for wound healing include medical device dressings and hyperbaric or negative pressure oxygen therapy [38]. In recent medical research, there is still a shortage of oral medications that directly improve wound healing. Among the available oral medications, most of them play an adjunctive role, such as infection relief, nutrition, and pain management [39].

### 4.2. The Effects of NK-4

In Japan, NK-4 has been used as an oral therapeutic agent to promote wound healing. Previous studies have shown that interferon-gamma (IFN-γ) production by splenocytes can be enhanced by the oral administration of NK-4 to male BALB/c mice in which the splenocytes have been stimulated with lipopolysaccharide (LPS). This phenomenon may be related to the activation of T cells by IL-12 produced by macrophages [40]. Another study elucidates the underlying mechanisms of NK-4 for wound healing. This study demonstrates that NK-4 drives macrophage polarization toward an inflammatory M1-like phenotype to increase macrophage phagocytic activity in the tests using the human monocytic cell line THP-1. This study also shows that NK-4 has the potential for treating persistent inflammation in chronic wounds [41].

## 5. Antiviral Infection

### 5.1. Pathogenesis and Pharmacological Therapy

Viruses are non-cellular organisms composed of a nucleic acid molecule (DNA or RNA) and proteins. It can only synthesize its own nucleic acid and protein components by using the metabolic system in the host’s living cells and can only reproduce itself in large quantities. Under the condition of leaving the host cell, it can exist in the state of inanimate biological macromolecules and maintain its invasive and infectious viability for a long time. The nucleic acid of some viruses can also integrate into the genome of the host cell, then inducing latent infection [42]. 

According to the classification of strategies, antiviral drugs are mainly concentrated in two directions: targeting the virus itself or the host cytokines [43]. Based on the mechanism of antiviral drugs, the current antiviral drugs can be divided into the following categories: (1) preventing viruses from penetrating into the host cell and from inhibiting the uncoating (amantadine, rimantadine) [44]; (2) DNA polymerase inhibitors (acyclovir, ganciclovir) [45]; (3) nucleoside reverse transcriptase inhibitors (lamivudine, emtricitabine), and non-nucleoside reverse transcriptase inhibitors (efavirenz, nevirapine) [46,47]; (4) neuraminidase inhibitors (oseltamivir, zanamivir) [48]; (5) protein inhibitors saquinavir [49]; and (6) broad-spectrum antiviral drugs (interferon, ribavirin) [50].

### 5.2. The Effects of NK-4

Ushio et al. validated the antiviral effect of NK-4 and used herpes simplex virus (HSV)-1 to produce pathological effects on human amniotic fluid cells as a model. The experimental results showed that NK-4 had no direct inhibitory effect on HSV-1, but mainly due to an indirect effect mediated by fluid cells to reduce HSV-1 replication by a dose-dependent manner. Furthermore, NK-4 itself significantly induced the alkalinization of intracellular organelles, leading to the inhibition of viral entry into cells. NK-4 also enhances the antiviral effects of interferon (IFN)-α [51]. 

## 6. Treatment of Dilated Cardiomyopathy 

### 6.1. Pathogenesis and Pharmacological Therapy

Dilated cardiomyopathy (DCM) is a primary cardiomyopathy of unknown cause. It is characterized by the progressive enlargement and exacerbation of the left, right, or bilateral ventricles, leading to myocardial contractile dysfunction with or without congestive heart failure [52]. Death from dilated cardiomyopathy can occur at any stage of DCM. Common causes of DCM include viral infection of cardiomyocytes, genetic inheritance, and autoimmune disease. 

Pathological factors for left ventricular expansion are mainly related to remodeling and fibrosis. The pathological mechanisms of DCM mainly include: (1) genetics: the most common genes responsible for DCM are *TTN*, *BAG3*, *TNNT2*, *MYH7*, *RBM20*, *LMNA44*, *PRDM16*, etc. [53]; (2) autoimmunity: immune cell infiltration, aberrant expression of adhesion molecules, or HLA II in the heart was found in 50% of biopsy samples. According to the Rose–Witebsky criteria, DCM may be caused by an autoimmune condition [54]; (3) infection: infectious factors (mainly myocarditis) account for approximately 30% of the pathophysiology of DCM. Common groups of viruses associated with DCM include parvovirus B19, enteroviruses, herpesviruses, and adenoviruses [55]; (4) inflammation: inflammation associated with autoimmunity and viral infection is involved in the pathogenesis of DCM. Myocardial biopsy specimens from patients with DCM showed that high expression of tenascin-C resulted in poor patient survival [56]; and (5) exposure to toxins and chemicals, such as metals (mercury, lithium, antimony, cobalt), scorpion venom, antidiabetic drugs, anticancer drugs, antiretroviral agents, cocaine, ethanol, methamphetamines, and carbon monoxide. The mechanisms of toxic cardiomyopathy include the production of reactive oxygen species (ROS), intracellular calcium handling, interference with mitochondrial respiration in cardiomyocytes, neurohormonal stress, genetic susceptibility, and apoptosis [57]. The main treatment methods for dilated cardiomyopathy include: (1) controlling blood pressure, improving blood flow, and reducing the burden on the heart. Antihypertensive drugs include angiotensin II receptor blockers (ARBs), angiotensin-converting enzyme (ACE) inhibitors, beta-blockers, and sacubitril; (2) controlling the heart rhythm, strengthening the contraction of the heart muscle, slowing down the heartbeat, and reducing the symptoms of heart failure, such as digoxin; (3) preventing blood clots and anticoagulants such as warfarin and direct oral anticoagulants; (4) reducing fluid in the body and improving dyspnea caused by pulmonary hypertension, such as from diuretics; and (5) surgery and heart transplantation [58].

### 6.2. The Effects of NK-4

**Figure 2:**
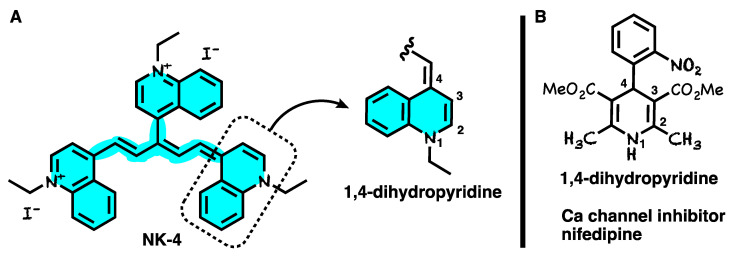
(**A**) Chemical structure of NK-4 having a 1,4-dihydropyridine moiety. (**B**) Structure of Ca channel inhibitor nifedipine having a 1,4-dihydropyridine moiety.

Transient receptor potential vanillin 2 (TRPV2) is a prime candidate for aberrant Ca^2+^ entry pathways and a potential target for the treatment of DCM [59]. NK-4 is one of the TRPV2 inhibitor candidates. Experiments demonstrate that low-dose NK-4 inhibits TRPV2 channel activity, which in turn inhibits abnormally increased Ca^2+^ influx, prevents the progression of DCM in dystrophic hamsters (J2N-k), and improves cardiac function [2]. It is noteworthy that the structure of NK-4 (Figure 2A) has a 1,4-dihydropyridine moiety-like structure which is similar to a representative Ca channel inhibitor, nifedipine (Figure 2B). Thus, the TRPV2 channel inhibitory activity of NK-4 might be derived from the 1,4-dihydropyridine moiety.

## 7. Anti-Oxidative

### 7.1. Pathogenesis and Pharmacological Therapy

Oxidative stress is considered to be an imbalance between the oxidative stress and antioxidative systems of cells, leading to inflammatory infiltration of neutrophils, increased secretion of proteases, and the production of large amounts of reactive oxygen species (ROS) and reactive nitrogen species (RNS). In particular, oxygen free radicals can cause damage to the phospholipids, proteins, enzymes, and nucleic acids of cells, resulting in cell dysfunction. There are many markers of oxidative stress, including lipid hydrogen peroxide, isoprostanes, and more [60,61,62]. 

According to the solubility, antioxidants are divided into oil-soluble (butylated hydroxyanisole (BHA) and butylated hydroxytoluene (BHT)) [63] and water-soluble (tea polyphenols, ascorbic acid, ascorbyl palmitate) [64,65,66]. Antioxidants can also be divided into synthetic antioxidants (butylated hydroxyanisole (BHA); butylated hydroxytoluene (BHT); propyl gallate (PG); tertiary butylhydroquinone (TBHQ)) [67] and natural antioxidants (caffeic, rosmarinic acids, carnosol, quercetin, eugenol, anthocyanin) [68,69]. On the basis of the mechanism of action of antioxidants, antioxidants are classified into free radical scavengers [70], hydrogen peroxide scavengers [71], metal chelating agents [72], enzymatic and non-enzymatic antioxidants [73], and singlet oxygen quenchers [74].

### 7.2. The Effects of NK-4

**Figure 3:**
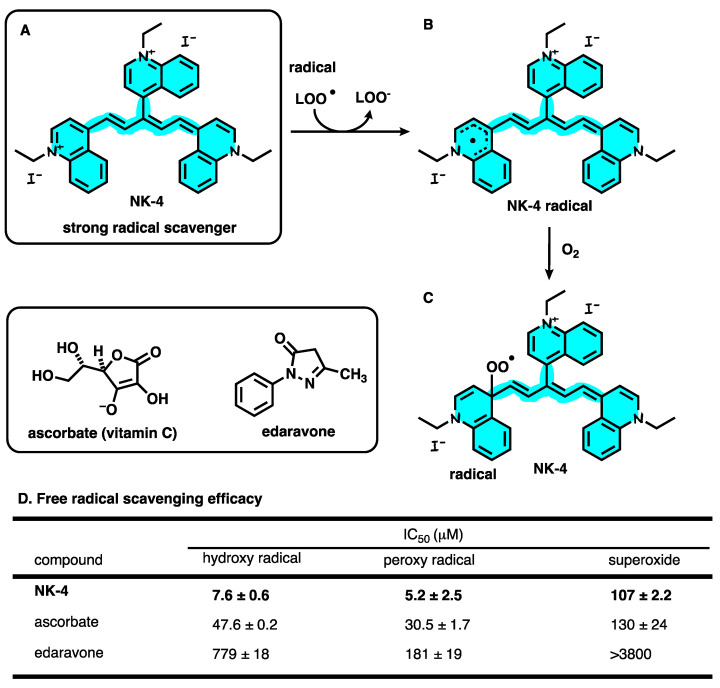
(**A**) Chemical structure of NK-4. (**B**) Structure of NK-4 radical. (**C**) NK-4 peroxy radical generated by O_2_. (**D**) Free radical scavenging efficacy.

Previous studies have shown that NK-4 has been tested to have significant hydroxyl radical scavenging activity, whereas NK-4 has also been demonstrated to be effective at scavenging peroxyl radicals in vitro by electron spin resonance (ESR) techniques [75]. In vivo, NK-4 was intravenously injected twice in an animal model of ischemic stroke (the middle cerebral artery occlusion (MCAO) model rat), which was induced by the temporary ligation of middle cerebral artery, followed by the reperfusion. NK-4 reduced the infarct volume by 57.0%. The results demonstrate that NK-4 can prevent cerebral ischemic injury and reduce cerebral ischemic damage by reducing reactive oxygen species (ROS), including superoxide (•O_2_^−^), hydroxyl radicals (•OH), and hydrogen peroxide (H_2_O_2_) (Figure 3A–C). NK-4 also reduces ischemic swelling of the brain hemispheres [75,76]. Another study evaluated the protective effects of NK-4 on oxidatively damaged nerves in vitro and in vivo. In vitro, NK-4 has free radical-scavenging activity by means of clearing hydroxyl radical, peroxy radical, and superoxide. The studies which compared NK-4 with other neuroprotectants showed that NK-4 has significantly higher hydroxyl radical scavenging activity than ascorbic acid and edaravone (Figure 3D). In vivo, the activation of PI3K and its downstream signaling effector Akt may be designated as a key mediator system that is beneficial to neuronal survival by NK-4 injection, and the antioxidant properties of NK-4 may also be associated with neuronal survival and functional maintenance [77]. In a recent study, researchers injected NK-4 into the eyes of Royal College of Surgeons rats (a model rat of retinitis pigmentosa) that exhibit inherited retinal dystrophy. The results show that NK-4 delays photoreceptor apoptosis through anti-oxidation, the maintenance of intracellular ion homeostasis, and other mechanisms [78].

## 8. Neuroprotective Effects

### 8.1. Pathogenesis and Pharmacological Therapy

Neurodegenerative diseases are caused by the loss of neurons, myelin sheaths, and synapses. Neurodegenerative diseases can be caused by aging and genetic mutations, and the condition of the diseases worsens over time, leading to functional impairment [79]. Common pathogenic mechanisms of neurodegenerative diseases include: (1) abnormal protein dynamics (protein misfolding and aggregation); (2) oxidative stress (formation of reactive oxygen species and free radicals); (3) dysfunction of neurotrophic factors; (4) mitochondrial dysfunction; (5) neuroimmune inflammation; (6) neuronal Golgi breakdown; (7) disruption of cell/axon transport; and (8) altered cell signaling. Altogether, the diversity of multiple pathogenic factors leads to multifaceted neuronal death [80]. 

The main research areas of neurodegenerative diseases include: (1) tau protein disease—Alzheimer’s disease (AD); (2) extrapyramidal disorder: Parkinson’s disease (PD), Huntington’s disease (HD); (3) spinocerebellar degeneration: multiple system atrophy (MSA); (4) autonomic disorders: Shy-Drager syndrome (SDS); and (5) motor neuron disorders: amyotrophic lateral sclerosis (ALS), Werdnig–Hoffmann disease. Ophthalmological neurodegenerative diseases mainly include retinitis pigmentosa (RP).

The main drugs for neurodegenerative diseases include: galantamine, rivastigmine, and donepezil for Alzheimer’s disease [81,82,83]; levodopa, monoamine oxidase-B inhibitors, and dopamine agonists for Parkinson’s disease [84]; tetrabenazine (Xenazine) and deutetrabenazine (Austedo) for Huntington’s disease [85]; fingolimod (Gilenya), dimethyl fumarate (Tecfidera), and teriflunomide (Aubagio) for multiple sclerosis (MS) [86]; and Radicava, rilutek, exservan, nuedexta, and tiglutik for amyotrophic lateral sclerosis [87]. As a therapeutic drug for retinitis pigmentosa, Luxturna^®^ (voretigene neparvovec) is the only Food and Drug Administration (FDA)-approved retinitis pigmentosa therapy, designated for a small subset of patients with *RPE65* mutations [88].

On 23 June 2022, the FDA published a 5-year action plan for drugs of neurodegenerative diseases, focusing on ALS [89,90]. Therefore, with the deepening of neurodegenerative disease research, multi-pathway and multi-target therapeutic drugs urgently need to be developed. 

### 8.2. The Effects of NK-4

In a report, besides the neurotrophic and neurogenesis activity of NK-4 observed in a transgenic mouse model of Alzheimer’s disease (Tg 2576), the effect of NK-4, which was better than acetylcholinesterase inhibitors (AChEIs), was also observed in the early stages of mouse dementia (6 months old). NK-4 may be a new drug for the treatment of early- to late-stage Alzheimer’s disease [91]. Another study showed that NK-4 had neurotrophin-like activity and exhibited neuroprotective effects in vitro and in vivo. In vitro, NK-4 significantly enhanced nerve growth factor (NGF)-induced neurite outgrowth in PC12HS cells. In vivo, NK-4 effectively prevented injury in a rat stroke model (middle cerebral artery occlusion (MCAO) Rats) through neurotrophin-like activity and antioxidative activity [75]. In vitro, NK-4 was shown to dose-dependently protect PC12 cells from oxidative stress-induced toxicity by 6-hydroxydopamine (6-OHDA) or hydrogen peroxide (H_2_O_2_). In an ataxia animal model (Syrian hamster marked by Purkinje cell degeneration, PCD model) of neurodegeneration, the studies showed that the neuroprotective effects of NK-4 are mediated by the PI3K-Akt signaling pathway [92]. NK-4 can also reduce the accumulation of Aβ in the brain, inhibit Aβ aggregation, scavenge free radicals, and produce neuroprotective effects by its intraperitoneal injection in Alzheimer’s disease model AβPP transgenic mice (Tg2576). It is thus suggested that NK-4 can also be used to treat Alzheimer’s disease [77,93].

In a recent study, researchers administered NK-4 into the eyes of RCS rats via intravitreal injection; the researchers found that NK-4 could inhibit the apoptosis of photoreceptor cells. *Hmox1*, *Mt1*, *Atf5*, *Slc7a11*, and *Bdh2* genes were up-regulated by the RNA-seq analysis and confirmed by the RT-PCR analysis. Functional and pathway enrichment analyses of up-regulated genes in that study suggest that the neuroprotective effect of NK-4 in RCS rat retina might be related to the retinal pigment epithelial metabolic process, transition metal ion homeostasis, and negative regulation of neurons’ apoptosis by Metascape analysis. They also uploaded five genes (*Hmox1*, *Mt1*, *Slc7a11*, *Bdh2*, *and Atf5*) to the DAVID database for the functional annotation clustering of bioinformatics resources. Based on the gene function distributed by DAVID, it was divided into the following categories: response to oxidative stress, negative regulation of neuron apoptotic process, and iron ion homeostasis [78]. All of these results revealed the molecular mechanism by which NK-4 inhibits the apoptosis of photoreceptor cells, indicating that NK-4 upregulates genes involved in anti-oxidative stress and anti-apoptotic pathways.

## 9. Synthesis of NK-4

**Figure 4:**
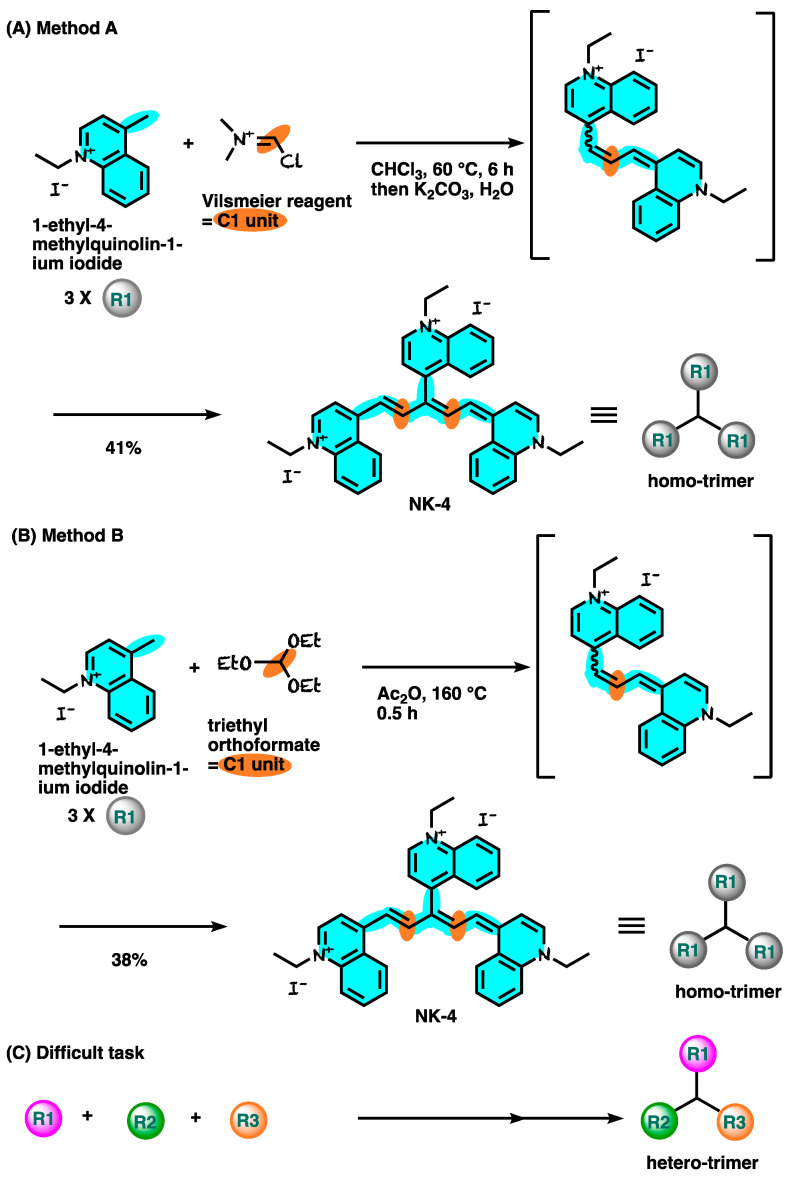
Synthetic route for NK-4.

The synthetic route for NK-4 is shown in Figure 4 [94,95]. 1-Ethyl-4-methylquinolin-1-ium iodide was treated with Vilsmeier reagent generated in situ from P(O)Cl_3_ and *N*,*N*-dimethylformamide, affording the desired NK-4 at a 41% yield (Figure 4A). A different C1 unit protocol was also reported (Figure 4B). However, these routes could be used for the same three-quinoline moiety, but not for hetero-quinoline moieties. In the future, the development of a new synthetic methodology is highly required to supply various derivatives which have different quinoline cores (Figure 4C).

## 10. Summary

In nearly 70 years of research, NK-4 has been developed for various pharmacological effects, including anti-inflammatory, anti-allergic, anti-cancer, wound healing, antiviral, antioxidative, and neuroprotective effects. NK-4 is a good candidate for treating various diseases, and it is expected that the pharmacological properties of NK-4 can be applied to the treatment of many more diseases, such as neurodegenerative and retinal degenerative diseases. With respect to the antioxidative effect, NK-4 has a higher hydroxyl radical scavenging activity compared with other antioxidants. To plan experiments for assessing the antioxidative and neuroprotective effects of NK-4, more animal models are needed to verify these effects and the pharmacological mechanisms of NK-4, and to proceed towards the goal of successfully entering clinical trials. Overall, this review provides a summary of the various functions of NK-4 as insights for the development of potential therapeutic agents.

## Data Availability

Not applicable.
